# Retraction: PTIP inhibits cell invasion in esophageal squamous cell carcinoma *via* modulation of EphA2 expression

**DOI:** 10.3389/fonc.2026.1881179

**Published:** 2026-05-28

**Authors:** Frontiers Editorial Office

The publisher retracts the article cited above.

Following the publication of the article, the authors contacted the Editorial Office to request a correction of the cited article after a comment on PubPeer reported image duplication. An investigation was conducted in accordance with Frontiers’ policies, during which the authors requested the retraction of the article, citing that they had discovered significant errors in the data post-publication. Upon applying the corrected data, the results no longer supported the original conclusions.

Given the serious concerns regarding the validity of the data, the editors no longer have confidence in the findings presented in the article.

This retraction was approved by the Chief Editors of Frontiers in Oncology and the Chief Executive Editor of Frontiers. The authors were formally notified of the retraction and were given an opportunity to respond. The publisher has documented this communication.

Frontiers would like to thank Sholto David for bringing the published article to our attention.

